# Fatal Course of Abdominal Neonatal Intestinal Fibrosarcoma

**DOI:** 10.1055/s-0039-1692154

**Published:** 2019-06-09

**Authors:** Béatrice Boutillier, Liesbeth Cardoen, Marianne Alison, Dominique Berrebi, Jonathan Rosenblatt, Anne-Laure Virlouvet, Jean Michon, Sophie Soudée, Arnaud Bonnard

**Affiliations:** 1 Department of Radiology, Robert-Debré University Hospital, Assistance-Publique Hôpitaux de Paris, Paris, Île-de-France, France; 2 Department of Anatomopathology, Robert-Debré University Hospital, Assistance-Publique Hôpitaux de Paris, Paris, Île-de-France, France; 3 Department of Gynecology and Obstetrics, Robert-Debré University Hospital, Assistance-Publique Hôpitaux de Paris, Paris, Île-de-France, France; 4 Department of Neonatology and Intensive Care Unit, Robert-Debré University Hospital, Assistance-Publique Hôpitaux de Paris, Paris, Île-de-France, France; 5 Department of Pediatric Oncology, Institut Curie, Paris, Île-de-France, France; 6 Department of Pediatric Surgery and Urology, Robert-Debré University Hospital, Assistance-Publique Hôpitaux de Paris, Paris, Île-de-France, France; 7 UMR 1149 Inserm Universite Paris Diderot, Sorbonne Paris Cité, Paris, Île-de-France, France

**Keywords:** congenital fibrosarcoma, prenatal diagnosis, MR Imaging, haemorrhagic shock, meconium peritonitis

## Abstract

Infantile fibrosarcoma (IFS) is a rare nonrhabdomyosarcoma soft tissue tumor and accounts for less than 1% of childhood cancers. Forty per cent are present at birth and only 10% of IFS occurs in the abdomen. Our case of neonatal fibrosarcoma presented as a distal small bowel stenosis complicated with meconium peritonitis. The diagnosis was by histology of the surgical resection. The diagnosis of IFS is challenging as there are no specific features of IFS on imaging. Any unexpected solid lesion should raise the suspicion of complicated bowel tumoral lesion. If a neoplastic lesion is suspected extensive, surgery may be postponed until the final diagnosis is made.

## Introduction

Infantile fibrosarcoma (IFS) is a very rare condition occurring in approximately five new cases per 1 million children. 1 IFS accounts for less than 1% of childhood cancers and for 24% of soft tissue sarcomas. This is a rare nonrhabdomyosarcoma soft tissue tumor which develops at the expense of connective tissue. IFS accounts for 5 to 10% of soft tissue sarcoma before 1 year. 2 The average age at diagnosis is 3 months but 40% of tumors are present at birth. 2 Only 10% of IFS occurs in the abdomen and few of them involve the gastrointestinal tract. 3 We present a case of neonatal intestinal fibrosarcoma diagnosed prenatally as a meconium cyst.

## Case Report

**Fig. 1:**
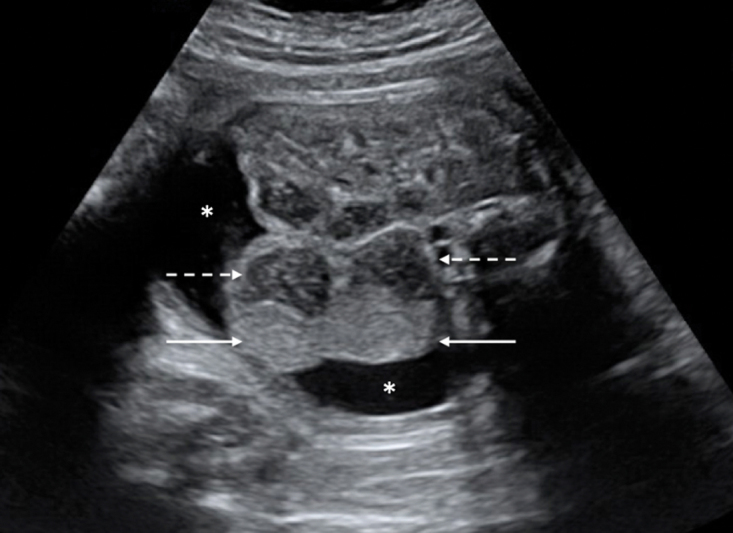
Prenatal ultrasound, transverse slice at the level just below the liver: visualization of a hyperechogenic mass (white solid arrows) adjacent to a dilated digestive loop (white dotted arrows), surrounded by anechogenic ascites (asterisk [*]) in the right hemiabdomen.

**Fig. 2:**
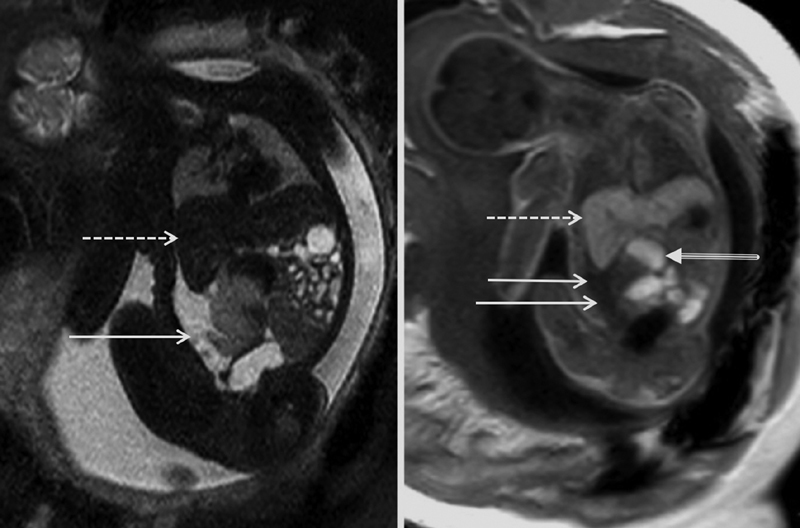
Fetal MRI, coronal slices (T2-sequence on the left side, T1 on the right side): the abdominal mass (white arrows) appears as an intermediate hyperintense T2-lesion and a hypointense to isointense T1-lesion. Visualization of some adjacent dilated bowel loops with meconial content (tri-line arrow). The dotted arrows show the liver. MRI, magnetic resonance imaging.

A child of unrelated parents was born at 37 gestation weeks (GW) by Caesarean section (weight 2,630 g). At 30 GW, fetal ultrasound (US) showed peritoneal ascites with septations and an echogenic mass adjacent to a right dilated bowel loop with an echogenic content ( Fig. 1 ). Antenatal magnetic resonance (MR) imaging at 33 GW confirmed the diagnosis ( Fig. 2 ) and demonstrated normal jejunal loops in the left flanck. This prenatal imaging was compatible with a bowel atresia, Meconium peritonitis (MP), and associated pseudocyst (cystic meconium peritonitis).

The prenatal investigation of the amniotic fluid was normal (karyotype and digestives enzymes) and cystic fibrosis was ruled out. At birth, the abdomen was distended. Contrast enema demonstrated a micro colon. The US revealed echogenic ascites with septations, a large epigastric dilated digestive loop with echogenic content, surrounded by a large, hyperechoic mass, with slight vascularization on the Doppler. At surgery, MP with a small bowel stenosis (25 cm proximal to the ileocaecal junction) was confirmed along with a soft tissue mass adjacent to the liver and the posterior peritoneum. Taken together, these observations suggested the presence of a meconium cyst. The mass was resected with difficulties related to the close relation with the liver, and anastomosis was performed. Due to extensive adhesiolysis, the resection was complicated by a haemorrhagic shock and required a cardiopulmonary resuscitation (37 minutes and 10 adrenaline doses). Due to extensive adhesiolysis, the resection was complicated by a hemorrhagic shock, that was characterized by a hemoglobin level lower than 8 g/dL, and below normal coagulation factor levels (platelets, aPTT [activated partial thromboplastin time], and factors V, and VIII) that barely reached 30% of the expected values. We performed a cardiopulmonary resuscitation that lasted 37 minutes and required the injection of 10 adrenaline doses and the transfusion of 100 mL/kg of packed red blood cells (PRCB), platelets, and 2 units of fresh frozen plasma (FFP). Despite medical treatment, including the administration of albumin and vasoactive drugs, the noncontrolled bleeding persisted, requiring a second surgery to place a perihepatic packing. It was removed 5 days later.

**Fig. 3:**
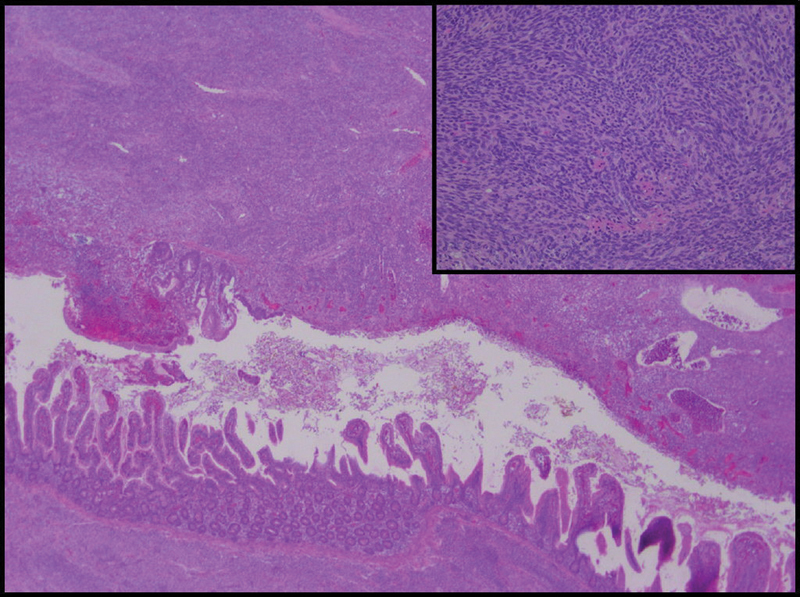
Intestinal fibrosarcoma (hematoxylin and eosin: original magnification ×25 and ×250 in inset), dense spindle cells proliferation occurring in the submucosa and extending to the whole intestinal wall.

The histopathologic examination revealed a dense cellular tumor in the ileal intestinal wall with spindle-shaped cells in clusters from submucosa to subserosa. The tumor cells had oval nuclei and blurred cytoplasmic limits with high mitotic activity and frequent apoptosis. They were arranged in interlacing fascicles following a characteristic herringbone pattern. The blood vessels were sheathed in the tumor and their walls were thickened. Moreover, inside the segment named “meconium cyst,” there was a lumen which was full of meconium and bordered by an intestine mucosa infiltrated with tumor cells meaning that the “cyst” was a part of the tumor ( Fig. 3 ).

Immunohistochemistry showed that tumor cells only expressed vimentin, to the exclusion of markers like desmin, catenin, and transmembrane phosphoglycoprotein protein encoded by the CD34 gene (cluster of differenciation 34 founded in hematopoietic cells) (CD34). Many nuclei expressed K _i_ -67. Cytogenetic examination with fluorescence in situ hybridization (FISH) analysis did not confirmed a rearrangement of the ETV6 region, and an translocation of genetic material between the *ETV6* gene located on the short arm of chromosome 12 at position p13. and the *NTRK3* gene located on the long arm of chromosome 15 at position q25.3 to create the (12;15)(p13;q25) fusion gene, ( *ETV6-NTRK3* ) fusion transcript was not detected using Reverse Transcriptase - Polymerase Chain Reaction (RT-PCR).

Because untreatable and fatal neurological sequelae were diagnosed, secondary to the hemorrhagic shock, palliative care was provided to the patient who died at 63 days of life.

## Discussion

The interest of this case lies in its location and its diagnostic timeline.

## Clinical Presentation

In 71% of the cases, IFS develops in the limbs. Nevertheless, several ectopic cases were observed, particularly in the trunk 4 5 or the neck. 4 Some of the described axial cases are located in the abdominal region and derive from the intestine. 3 They are associated with either a digestive perforation or a bowel obstruction. Some of the described axial cases are located in the abdominal region and derive from the intestine. 3 The study of the Berrebi cohort show that abdominal IFS are frequently associated with either a MP complicating a digestive perforation on the mesenteric side of the tumor wall (54% of the cases), or a bowel obstruction (30% of the cases). 3 A big mass could lead to a perforation and stenosis that is susceptible to hide the primitive cause and thus complicate the diagnosis. In our case, the cyst was closely related to the liver; therefore, the surgical dissection and separation of the cyst from the surrounding structures were extensive and leading significant blood loss and ultimately hemorrhagic shock. This finding must make think to another diagnosis than a usual meconium cyst related to an atresia.

When IFS is located in the abdomen, differential diagnosis, such as all intra-abdominal tumors, any remainder malignant mesenchymal tumors or sarcomas, 2 hemangioma or infantile fibromatosis, 2 and Meckel's diverticulum, 3 5 should be ruled out first. In our case, all the difficulties came from the unknown presurgical diagnosis.

## Imaging

On imaging IFS have nonspecific features. On US, they usually appear as a heterogeneous, well-defined soft tissue single mass, 4 6 most often hypoechogenic with an anechoic part (cystic). 4 On the Doppler, the blood flow is increased, especially at the periphery. 4 Magnetic resonance imaging (MRI) is the imaging of choice. The expected imaging results for IFS are well-defined heterogeneous soft tissue mass, with T1-low signal and T2-hyper signal. 4 7 8 Sometimes an hemorrhagic or necrotic content can be observed. 4 Internal fibrous septa have also been described. 7 High-flow vascular structures can be seen and after contrast injection, intense enhancement is usually depicted, which can be heterogeneous and predominant at the periphery. 4 8 However, we found a heterogeneous mass to the exclusion of any other criteria. This nonspecific observation led us to diagnose a meconial cyst.

In our case, the MP associated to a distal bowel atresia was misleading and the mesenteric lesion visualized on imaging was not taken into consideration at first, despite the Doppler showed a slightly increased vascularization but without preferential location and an heterogeneous mass which evoked a solid lesion. Fetal MRI was not helpful and postnatal MRI was not performed. Perhaps additional MRI should be performed when the diagnosis is ambiguous which was not done.

## Genetic and Anatomopathology

Pathological analyses of IFS show a tumoral lesion with regular walls, made of layers of closely packed spindle-shaped cells arranged in bundles and fascicles, resulting in a herringbone pattern. The mitotic activity is high and the nucleus is hyperchromatic. The vascularization is rich, and it is possible to find hemorrhagic, necrotic, or inflamed areas. 4 7 In immunohistochemistry, tumor cells express only vimentin whereas muscular markers, such as desmin, are negative 4 9 as found in our clinical report.

The ETV6-NTRK3 fusion transcript (FT) associated with the t(12;15)(p13;q25) translocation is a good biomarker for diagnosis, and allowed us to differentiate IFS from adult fibrosarcoma, infantile fibromatosis, and other tumors with spindle cells.

The genetic tests did not reveal the presence of an ETV6-NTRK3 fusion transcript, which doesn't mean it was absent because this test has a 9% false negative rate 9 . Given the consistency of the above listed histological criteria, this finding didn't alter our diagnosis.

## Treatment and Prognosis

The main treatment of neonatal IFS is the surgery with resection with as wide margins as possible, except if there is a risk of mutilation, in which case chemotherapy is used first. IFS indeed is a chemosensitive tumor that can be treated by the association vincristine-actinomycin (VA), a combination that demonstrated its efficacy with a response rate of 71%. 1 2

The global survival rate varies between 80 to 100%. 9 Metastases are rare (less than 5% of cases) and mainly axial (localized on the trunk). 2 The main risk is local relapse, 2 9 with a rate between 5 and 40% according to groups and localization. 9

In our case, no alternative therapeutic could be considered because we never thought about a differential tumoral diagnosis. During the surgery, as the tumoral diagnosis never mentioned, the surgeon thought removing a cyst, usual complication about meconial peritonitis; thus, the surgery is the only alternative and performing chemotherapy was not an option. Maybe we found in this example, a reason of the wait and see approach before 3 months, because of the dramatic outcomes of the surgery while the tumoral evolution is not aggressive. However, the surgery was required because of the bowel stenosis. After surgery, chemotherapy was not an option since the cerebral lesions were too important

## Conclusion

Cystic meconium peritonitis (CMP) is a result of in-utero bowel perforation. It will lead to secondary inflammation to the peritoneal cavity resulting in fibrosis, calcification, and cyst formation. Difficult surgery is expected as a result of the fibroadhesion and inflammation. Any unexpected solid lesion should raise the suspicion of complicated bowel tumoral lesion. If a neoplastic lesion is suspected extensive surgery may be postponed until the final diagnosis is made

## Notes

New Insights and the Importance for the Pediatric Surgeon We present a brief report describing a rare case of neonatal fibrosarcoma in an unusual localization diagnosed prenatally as a meconium cyst. The surgery was complicated by hemorrhagic shock and it highlights the importance of combining prenatal and postnatal imaging and genetic test to try to reach the diagnosis.
